# Biogenic Synthesis of Silver Nanoparticles and Their Diverse Biomedical Applications

**DOI:** 10.3390/molecules30153104

**Published:** 2025-07-24

**Authors:** Xiaokun Jiang, Shamma Khan, Adam Dykes, Eugen Stulz, Xunli Zhang

**Affiliations:** 1 Faculty of Engineering and Physical Sciences, University of Southampton, Southampton SO17 1BJ, UK; 2 Meinig School of Biomedical Engineering, Cornell University, Ithaca, NY 14853, USA

**Keywords:** biogenic synthesis, nanoparticles, silver nanoparticles, mechanisms of biogenic synthesis, biomedical applications

## Abstract

Nanoparticles (NPs) synthesised through biogenic routes have emerged as a sustainable and innovative platform for biomedical applications such as antibacterial, anticancer, antiviral, anti-inflammatory, drug delivery, wound healing, and imaging diagnostics. Among these, silver nanoparticles (AgNPs) have attracted significant attention due to their unique physicochemical properties and therapeutic potential. This review examines the biogenic synthesis of AgNPs, focusing on microbial, plant-based, and biomolecule-assisted approaches. It highlights how reaction conditions, such as pH, temperature, and media composition, influence nanoparticle size, shape, and functionality. Particular emphasis is placed on microbial synthesis for its eco-friendly and scalable nature. The mechanisms of AgNP formation and their structural impact on biomedical performance are discussed. Key applications are examined including antimicrobial therapies, cancer treatment, drug delivery, and theranostics. Finally, the review addresses current challenges, such as reproducibility, scalability, morphological control, and biosafety, and outlines future directions for engineering AgNPs with tailored properties, paving the way for sustainable and effective next-generation biomedical solutions.

## 1. Introduction

### 1.1. Background: Nanoparticles in Biomedical Applications

The integration of nanotechnology into medicine offers potential solutions to longstanding challenges in pharmacokinetics and therapeutic efficacy. Nanoparticles (NPs), ranging from 1 to 100 nanometres, have become pivotal in enhancing drug solubility, stability, and targeted delivery, minimising systemic toxicity and improving patient outcomes [1,2]. Since the late 20th century, synthetic NPs, produced via chemical methods like emulsification or physical techniques such as laser ablation, have dominated the field. Liposomes, polymeric NPs, and metallic NPs (e.g., gold, silver) have been extensively explored for their ability to encapsulate diverse payloads, including small molecules, proteins, and nucleic acids [3]. A landmark achievement was the FDA approval of liposomal doxorubicin (Doxil^®^) in 1995, which reduced cardiotoxicity, demonstrated improved pharmacokinetics in cancer patients compared to free doxorubicin [4]. Subsequent innovations, such as paclitaxel-loaded polymeric NPs, have further advanced treatments for cancers, infections, and chronic diseases by leveraging the enhanced permeability and retention (EPR) effect in diseased tissues [5].

However, synthetic NP synthesis often involves toxic solvents, high energy consumption, and complex purification, raising concerns about scalability, cost, and environmental impact [6,7]. For instance, chemical synthesis of gold NPs may leave residual reducing agents like sodium borohydride, posing risks of cytotoxicity [8]. These limitations have driven a shift toward sustainable alternatives. As the global nanomedicine market continues to expand, a growing emphasis is being placed on eco-friendly and biocompatible systems [9]. This evolution reflects the need for drug delivery platforms that balance efficacy with safety and environmental responsibility, setting the stage for greener nanotechnology approaches.

### 1.2. Emergence of Biogenically Synthesised Nanoparticles

Biogenically synthesised nanoparticles represent a transformative leap in this landscape, utilising biological entities, including plants, microorganisms, and biomolecules, to synthesise NPs with minimal ecological footprints. Unlike conventional synthetic methods, biogenic synthesis employs natural reducing and stabilising agents, such as plant-derived polyphenols, bacterial enzymes, or fungal metabolites, eliminating hazardous chemicals [10,11]. Early studies in the 2000s demonstrated that extracts from *Azadirachta indica* (neem) could produce silver NPs with antimicrobial properties rivalling chemically synthesised counterparts, but with lower toxicity [12]. Similarly, *Escherichia coli* and *Aspergillus niger* have been harnessed to synthesise gold and silver NPs via enzymatic reduction, offering scalable, green alternatives [13].

Recent advancements have significantly expanded the scope of biogenically synthesised nanoparticles (NPs). For example, biohybrid nanomaterials, comprising bioderived turmeric and silver/silver chloride nanoparticles, have been characterised for their antitumoral activity, demonstrating enhanced bioavailability and anticancer efficacy [14]. Microbial synthesis has also progressed, with AgNPs derived from the cyanobacterium *Spirulina platensis* showing promising antibacterial potential [15]. Biomolecule-based approaches, such as those using chitosan or albumin, offer precise control over NP size and surface chemistry, thereby improving drug loading capacity and targeting efficiency [16,17]. These NPs often exhibit inherent biocompatibility due to their biological coatings, e.g., proteins or polysaccharides, reducing immune responses and enhancing cellular uptake [18]. Furthermore, biogenically synthesised AgNPs are gaining attention in clinical research, with plant-derived NPs being explored in trials for wound healing and cancer therapy [19]. This shift highlights a broader trend toward sustainable nanomedicine, driven by eco-friendliness, cost-effectiveness, and strong therapeutic potential.

### 1.3. Scope and Objectives of the Review

This review aims to provide a comprehensive overview of the biogenic synthesis of AgNPs, specifically focusing on their microbiological synthesis routes, physicochemical properties, and biomedical applications. While recent developments in the literature on the biogenic synthesis of AgNPs are reviewed, the mechanistic fundamentals of silver nanoparticle formation and process optimisation are also examined. It explores three major biogenic synthetic pathways, including plant-mediated, microbial and biomolecule-based approaches with particular emphasis on microbial synthesis, highlighting how reaction conditions such as pH, temperature, media composition, and incubation time influence nanoparticles morphology, size, and surface chemistry. The review specifically highlights microbial synthesis due to its notable advantages in scalability and eco-friendly production.

The review also investigates the intracellular and extracellular mechanisms underlying AgNP formation, including enzymatic and electron transfer pathways. Furthermore, it discusses how the size, shape, and crystallographic facets of AgNPs impact their antimicrobial and therapeutic performance. Biomedical applications such as antibacterial treatments, cancer therapy, drug delivery, and synergistic effects with antibiotics are evaluated. Furthermore, the review critically examines current challenges associated with microbial synthesis, such as issues of reproducibility, standardisation, and biosafety. It concludes by outlining future research directions aimed at optimising microbial synthesis methods to achieve targeted and sustainable biomedical applications of silver nanoparticles.

## 2. Biogenic Synthesis of AgNPs

Biogenically synthesised NPs are nanoscale materials produced using biological entities such as plants, microorganisms, or biomolecules, distinguishing them from NPs synthesised via chemical or physical methods. Typically ranging from 1 to 100 nanometres, these NPs exhibit unique physicochemical properties, particularly functional surface groups, that make them promising candidates for biomedical applications [20]. Unlike chemically or physically produced NPs, biogenically synthesised NPs are often inherently biocompatible and biodegradable due to their biological origin, thereby reducing the risk of in vivo toxicity. Their surfaces may also carry natural capping agents, such as proteins or polysaccharides, which enhance colloidal stability, promote interactions with biological systems, and can sometimes direct the formation of anisotropic structure [21]. These characteristics position biogenically synthesised NPs as eco-friendly and functionally versatile alternatives in nanomedicine.

### 2.1. Biological Sources

The preparation of biosynthetic NPs can be achieved through various biological sources, including plants, microorganisms, and biomolecules. Plants are rich in diverse compounds such as carbohydrates, lipids, vitamins, proteins, flavonoids, and terpenoids, which have been demonstrated to play synergistic roles such as reducing agents, capping agents, and stabilisers in the synthesis of AgNPs [12,22]. In particular, plant-derived proteins exhibit excellent interfacial binding capabilities, enabling precise control over the morphology and size of nanoparticles while effectively inhibiting their aggregation and precipitation [23]. Additionally, this synthesis route demonstrates superior biocompatibility and safety, providing a reliable foundation for the biomedical applications of AgNPs [12,24].

Microorganisms, such as bacteria (*Escherichia coli*), fungi (*Aspergillus niger*), and algae (*Chlorella vulgaris*), synthesise NPs through enzymatic pathways or extracellular secretions [13]. The process of microbial synthesis of NPs can be divided into intracellular synthesis and extracellular synthesis. In intracellular synthesis, positively charged silver ions bind to the negatively charged microbial cell walls, where enzymatic reduction leads to NP formation and eventual release into the extracellular environment. In the extracellular pathway, metal ions interact with the outer microbial surface and are enzymatically reduced into nanoparticles in the surrounding medium [25].

Biomolecules, including proteins, enzymes, and polysaccharides, also facilitate NP synthesis by acting as templates, capping agents, or reducing agents [20]. Each biological source imparts distinct characteristics to the resulting nanoparticles, influencing their size, shape, stability, and functional properties.

### 2.2. Routes of Biogenic Synthesis

The synthesis of AgNPs has advanced considerably in recent years, with green chemistry approaches gaining prominence due to their eco-friendly and sustainable nature. These methods utilise biological entities as mentioned above, including mainly plants, microorganisms, and biomolecules, to mediate the reduction and stabilisation of metal ions into AgNPs. Below, these three key biosynthesis strategies are further explored, incorporating recent advancements and relevant references.

#### 2.2.1. Plant-Mediated Synthesis

Plant-mediated synthesis explores the rich reservoir of bioactive compounds found in various plant parts, such as leaves, roots, stems, flowers, and fruits, to facilitate the reduction of silver ions into AgNPs. This method is characterised by its simplicity, cost-effectiveness, and the elimination of hazardous chemicals, aligning well with the principles of green nanotechnology. For instance, Ahmed et al. [12] demonstrated that AgNPs synthesised using *Azadirachta indica* (neem) leaf extract exhibited a typical size distribution of 10–50 nm and showed potent antimicrobial activity against pathogens such as *Escherichia coli* and *Staphylococcus aureus*. In this process, phytochemicals, including flavonoids, terpenoids, and phenolic compounds, acted as both reducing and capping agents, thereby enhancing nanoparticle stability.

Recent studies have further refined this approach. For example, Gautam et al. [26] explored the use of *Ocimum sanctum* (holy basil) extracts for AgNP synthesis and reported enhanced antibacterial activity against clinically isolated multidrug-resistant *Acinetobacter baumannii*, attributed to the high eugenol content in the extract. Similarly, AgNPs biogenically synthesised using green tea extract showed improved anticancer activity and reduced cytotoxicity towards melanoma and normal murine cell lines, highlighting the versatility of plant-mediated synthesis methods [27].

These advancements underscore the potential for scalability and reproducibility in plant-mediated synthesis. However, challenges remain in standardising extract composition due to variability across plant species, geographical sources, and seasonal changes. Nevertheless, the absence of toxic solvents and the reliance on renewable resources position this approach as a sustainable and promising method for AgNP production.

#### 2.2.2. Microbial-Based Synthesis (Bacteria, Fungi, Algae)

Microbial synthesis exploits the metabolic capabilities of microorganisms, bacteria, fungi, and algae, to produce AgNPs with tailored properties. Bacteria such as *Bacillus subtilis* can reduce metal salts like silver nitrate either extracellularly, via secreted enzymes, or intracellularly, within the cell membrane [13]. Fungi, such as *Fusarium oxysporum*, are particularly valued for their ability to secrete reductive enzymes like nitrate reductase, which facilitate AgNPs formation with high yields [28]. Algae, enriched with pigments such as chlorophyll and fucoxanthin, offer another promising avenue. For instance, unicellular microalgae *ulvophyte* sp. has been used to synthesise AgNPs demonstrating anticancer and antibacterial effects, as reported by Hamida et al. [29]. These algal NPs benefit from the natural capping agents present in algal biomass, reducing aggregation.

However, microbial synthesis faces hurdles in scalability and cost. The need for controlled culture conditions, such as pH, temperature, and nutrient supply, can complicate large-scale production. Recent efforts have focused on optimising these parameters. For example, studies have been conducted on process optimisation for green synthesis of AgNPs for enhanced antibacterial properties [30], and thorough examination of the culture conditions mentioned above [21]. Although progress has been made in process control, microbial synthesis strategies still need to be further optimised to enhance their competitiveness compared to traditional chemical synthesis methods at industrial scale.

#### 2.2.3. Biomolecule-Based Synthesis (Proteins, Enzymes, Polysaccharides)

Biomolecule-based synthesis employs purified biological molecules such as proteins, enzymes, and polysaccharides to direct the assembly of NPs achieving precise control of particle size, morphology, and function. Proteins like albumin or enzymes such as laccase serve as reducing agents by donating electrons to metal ions, while polysaccharides like chitosan or alginate stabilise the resulting NPs by preventing aggregation [20]. Additionally, naturally occurring humic ligands complex organic substances derived from humic substances have gained attention for their dual role as both reducing and stabilising agents in AgNPs synthesis. Their diverse functional groups, such as carboxyl and phenolic hydroxyl moieties, contribute to effective metal ion reduction and nanoparticle capping, enhancing colloidal stability and biocompatibility [31].

Recent research has expanded this field significantly where chitosan-stabilised AgNPs exhibited enhanced biocompatibility and antimicrobial activity, making them suitable for biomedical applications [16]. Overall, this method’s strength lies in its ability to tailor NP characteristics for specific applications, such as drug delivery or catalysis. However, it is constrained by the availability and cost of purified biomolecules, which can limit scalability. Advances in recombinant DNA technology and bioprocessing may mitigate these issues [32].

To summarise the characteristics of the three biosynthetic methods described above, their advantages, disadvantages, and differences in formed nanoparticles are presented in Table 1.

### 2.3. Process Optimisation of Biogenetic Synthesis

Biosynthetic reaction conditions play an essential role in the formation of AgNPs, as demonstrated by the pioneering discovery of *Pseudomonas stutzeri* AG259, the earliest bacterium reported to produce AgNPs [33]. Since then, numerous microbial species have been identified with similar capabilities, particularly in extracellular synthesis, where nanoparticles are formed outside the cell using culture supernatants or cell-free extracts. This approach offers advantages in nanoparticle recovery and scalability.

The biosynthesis process, however, is influenced by a complex interplay of chemical and physical factors, including thermodynamics, enzyme kinetics, and environmental catalysts, all closely tied to the host organism’s biology. For instance, while AgNP formation by *A. kerguelensis* remains unaffected by incubation temperature, *P. antarctica* shows enhanced nanoparticle stability at lower temperatures [34]. These observations highlight that optimal biosynthetic conditions are organism-specific and multifactorial while further work is performed to identify the parameters that enable efficient and stable nanoparticle synthesis.

#### 2.3.1. Incubation Time 

Incubation time plays a critical role in the biosynthesis of AgNPs, influencing both yield and particle characteristics. In *Trichoderma longibrachiatum*, synthesis began after 72 h, as indicated by a colour shift and UV–vis absorbance at 384 nm, consistent with surface plasmon resonance (SPR). Increased biomass led to higher nanoparticle yield without altering peak position or particle morphology, suggesting a uniform, extracellular, time-dependent reduction process [35]. By varying incubation time, different microbial systems can show varied responses. *Comamonas acidovorans* exhibited spectral peak broadening over 72 h, indicating polydispersity [36], while *Aeromonas* sp. SH10 showed red shifts over six days, suggesting particle growth [37]. In *Morganella psychrotolerans*, time- and temperature-dependent SPR patterns revealed faster and more uniform synthesis at 20–25 °C, whereas lower temperatures delayed formation and favoured larger or aggregated particles [38]. Similarly, *A. kerguelensis* maintained stable signals, while *P. antarctica* showed peak broadening, highlighting differing stabilities. Media-only controls showed faster signal decay, underscoring the stabilising role of microbial metabolites [34].

While the biosynthesis process using *Cupriavidus necator*, *Bacillus subtilis*, and *Bacillus megaterium* showed clear colour changes within hours, with intensity depending on temperature and pH, AgNPs formed with intracellular extracts appeared paler even after 27 h. This is in contrast to the richer coloration achieved with extracellular supernatants highlighting how time, combined with environmental and biological conditions, influences nanoparticle synthesis. These findings suggest incubation time significantly affects AgNP yield and may subtly influence morphology and stability, depending on the microbial system and surrounding conditions [39]. Yet, the exact interplay between time and particle features remains partially unresolved, requiring deeper investigation into how time-dependent dynamics can be harnessed for precise nanoparticle design.

#### 2.3.2. Media Composition

The composition of growth media plays a pivotal role in the biosynthesis of AgNPs, significantly impacting nanoparticle yield, size, morphology, and stability. Simpler or nutrient-limited media often lead to the production of smaller and less aggregated nanoparticles, likely due to the lower ionic strength and presence of fewer interfering components. For instance, although *E. coli* exhibits comparable biomass growth in both LB and Nutrient Broth (NB), AgNP synthesis is notably enhanced in LB, suggesting that specific media components modulate the reduction process [40].

Studies have demonstrated that individual constituents like peptone, beef extract, and NaCl possess limited reducing potential compared to complete media or bacterial cell-free supernatants (CFS), where the presence of secreted enzymes, biosurfactants, and other metabolites synergistically drive efficient nanoparticle formation. A clear demonstration of this was observed when silver nitrate was mixed with nutrient broth, producing only a faint UV–vis signal at 410 nm, whereas CFS from *A. kerguelensis* and *P. antarctica* yielded much stronger and more stable AgNP signals. Peptone exhibited the highest individual potential among the broth components, but none matched the stability and efficiency of bacterial supernatants. Notably, when *A. kerguelensis* supernatant was added to an ongoing AgNP reaction involving *P. antarctica* or nutrient broth, it arrested the decline in absorbance and maintained nanoparticle stability for up to 48 h, highlighting the critical role of biological factors in both synthesis and stabilisation [34,41].

Similarly, *Bacillus subtilis* T-1 cultured on brewery effluents, molasses, and LB medium also exhibited variable AgNP synthesis performance. UV–vis spectroscopy confirmed nanoparticle formation with characteristic peaks at 450–500 nm, while dynamic light scattering and TEM analyses revealed that brewery effluent supernatants produced the smallest and most stable nanoparticles (4–5 nm), compared to 50 nm particles from LB medium. These particles showed high zeta potential (−30 to −40 mV), indicating strong colloidal stability and reduced aggregation tendency [42].

Interestingly, media preferences vary across species. *Streptomyces rochei* favoured minimal media with low KNO_3_, in contrast to *E. coli* DH5-α, which showed enhanced AgNP synthesis in nitrate-rich broth, possibly due to upregulated nitrate reductase activity, an enzyme implicated in silver ion reduction. Furthermore, a variety of bacterial-derived products, including biosurfactants, exopolysaccharides, bioflocculants, and pigments, are increasingly recognised as key agents in nanoparticle mediation and stabilisation. Some bacteria are capable of producing both intracellular and extracellular AgNPs, with the mode of synthesis influenced by environmental conditions such as pH, temperature, and nutrient availability [40].

These findings highlight the critical interdependence between microbial physiology, growth media composition, and environmental parameters in dictating the efficiency and characteristics of biogenically synthesised AgNPs. Understanding these relationships is crucial for optimising green nanotechnology approaches in biomedical and environmental applications.

#### 2.3.3. Temperature

Temperature plays a crucial role in the biogenic synthesis of AgNPs, as it directly affects both the enzymatic reduction mechanisms and the thermodynamics of nanoparticle formation. Elevated temperatures increase the kinetic energy and reaction rates, particularly in cell-free extracts, thereby enhancing the chemical reduction of metal ions. However, excessively high temperatures may denature or deactivate key enzymes involved in the reduction process, negatively impacting nanoparticle yield [43]. Studies have shown that temperature requirements for silver nanoparticle (AgNP) synthesis vary among bacterial species. For instance, Aeromonas and Corynebacterium exhibit optimal AgNP production at 60 °C, while others such as *Escherichia coli* and *Yersinia enterocolitica* require elevated temperatures to initiate nanoparticle formation, suggesting a more chemically driven synthesis mechanism [37,44,45].

Comparative experiments using *E. coli*, *Klebsiella pneumoniae*, and *Bacillus cereus* at 37 °C and 50 °C further support this temperature dependence. These studies found that the production of both silver and gold nanoparticles was significantly more efficient at 50 °C, likely due to enhanced enzymatic activity at temperatures closer to the organisms’ optimal metabolic range.

The effect of temperature on AgNP synthesis was further studied using psychrophilic bacterial species cultured in salt-free media at 4 °C and 30 °C. UV–vis spectroscopy confirmed gradual nanoparticle formation over prolonged incubation [34]. Notably, synthesis yield and rate were significantly higher at 30 °C, with *Yersinia kristensenii* producing the highest yield, while Aeromonas *salmonicida* exhibited the lowest at 4 °C and displayed unique spectral characteristics. TEM analysis revealed that AgNPs synthesised at 30 °C were generally larger, with *A. salmonicida* producing the largest particles, whereas those formed at 4 °C were smaller and more uniform [46]. *Psychrobacter* sp. at 4 °C produced the most monodisperse nanoparticles, but the same species yielded highly polydisperse particles at 30 °C, underscoring the impact of temperature on particle uniformity [34].

In fungal-mediated synthesis using *Trichoderma longibrachiatum*, 28 °C was identified as the optimal temperature for AgNP production, indicated by a visible colour change and a strong UV–vis absorption peak at 385 nm [35]. No synthesis occurred at 23 °C or 33 °C, highlighting the sensitivity of fungal enzymatic systems to temperature variation. At lower temperatures like 10 °C, larger nanoparticles (80–100 nm) were produced, whereas smaller, more uniform particles (10–40 nm) formed at 27–28 °C [35]. These differences align with surface plasmon resonance shifts, where smaller particles absorb at shorter wavelengths.

Despite the clear advantage of higher temperatures in accelerating nanoparticle synthesis and improving yield, they do not necessarily ensure improved nanoparticle stability, an essential criterion for applications in antimicrobial and biomedical fields. Therefore, temperature optimisation must strike a balance between synthesis efficiency and the structural integrity of nanoparticles to ensure consistent performance and application viability.

#### 2.3.4. Aerobicity

The oxygenation state of the culture environment is a critical parameter in the biogenic synthesis of AgNPs, influencing not only enzymatic pathways but also the efficiency of nanoparticle formation. Aerobic conditions, commonly employed with agitation to enhance oxygen solubility, have been shown to significantly influence nanoparticle crystallinity and morphology [38,47]. In a study involving *Meyerozyma guilliermondii*, Ag/AgCl nanoparticle biosynthesis was evaluated under both aerobic and anaerobic conditions. X-ray diffraction (XRD) analysis of aerobically synthesised nanoparticles revealed sharp, well-defined peaks corresponding to both metallic silver (Ag^0^) and silver chloride (AgCl), indicative of a highly crystalline and phase-pure structure. In contrast, the anaerobic condition yielded XRD profiles with less intense and poorly defined peaks, suggesting lower crystallinity and potential morphological shifts toward amorphous or spherical structures [48].

However, some emerging evidence supports the efficacy of anaerobic conditions in enhancing AgNP synthesis in certain bacterial systems. For instance, Escherichia coli demonstrated superior AgNP production efficiency under anaerobic conditions, attributed to the upregulation of key reductive enzymes in the absence of oxygen. Central among these is nitrate reductase, an enzyme typically induced in anaerobic environments, which catalyses the reduction of Ag^+^ to Ag^0^ [49]. Studies on *Thiosphaera pantotropha* further corroborated this mechanism, showing that enhanced nitrate reductase activity directly facilitated efficient nanoparticle synthesis [49]. These findings highlight the central role of oxygen-sensitive enzymatic pathways in governing nanoparticle biosynthesis.

Additionally, microbial synergy has been explored as a means of optimising AgNP production. A mixed-culture approach using *Lactobacillus* sp. and *Bacillus* sp. under aerobic conditions yielded smaller, more uniformly dispersed, and highly crystalline nanoparticles compared to those produced by monocultures. This suggests that both microbial interactions and oxygen availability can be fine-tuned to control nanoparticle morphology, size distribution, and crystallinity [50].

In summary, both aerobic and anaerobic conditions exert profound effects on biogenic AgNP synthesis by modulating enzymatic activity and redox pathways. Strategic manipulation of oxygen levels in microbial cultures offers a viable route for tailoring nanoparticle properties to meet specific application requirements, particularly in biomedical, environmental, and catalytic domains.

#### 2.3.5. pH

Among environmental parameters affecting AgNP biosynthesis, pH is particularly crucial due to its impact on enzymatic activity. Most studies report optimal nanoparticle synthesis at near-neutral to alkaline conditions (pH 6–10), especially in extremophilic microbial systems [51,52]. For example, *Cupriavidus necator*, *Bacillus megaterium*, and *Bacillus subtilis* showed enhanced nanoparticle formation at pH 10, marked by intense brown coloration and characteristic UV–vis peaks, indicating higher yield and better stability. Acidic and neutral conditions, by contrast, resulted in poor or undetectable AgNP formation, likely due to reduced enzymatic efficiency and diminished reducing power [39,42,53].

A broader pH range study (pH 4–12) confirmed that basic environments facilitate Ag^+^ reduction by decreasing H^+^ ion concentration and promoting redox reactions. However, extreme alkalinity may lead to the formation of silver hydroxide (AgOH) or oxide precipitates, which reduce silver ion availability. This issue can be mitigated using alternative silver sources like Ag-amine complexes [51,54]. Some strains exhibit pH resilience. *A. kerguelensis* showed consistent AgNP production across pH 5–10, while *P. antarctica* displayed decreased nanoparticle stability at pH 7, with plasmon peaks diminishing over time, indicating aggregation [34]. Similarly, *Lactobacillus fermentum* showed optimal AgNP synthesis at pH 11.5, with higher silver recovery and faster reaction kinetics [55]. In contrast, acidic pH conditions generally suppressed AgNP formation, likely due to enzyme denaturation and reduced electron transfer efficiency.

Notably, some acidophiles such as *Corynebacterium* sp. and *Acidithiobacillus thiooxidans* effectively synthesise nanoparticles at low pH. At pH 2, *Corynebacterium* exhibited high Ag^+^ biosorption due to increased active binding sites on the cell surface, while uptake of [Ag(NH_3_)_2_]^+^ at pH 8 was enhanced due to stronger interactions with negatively charged surfaces [44].

To address pH-associated challenges, strategies include using pH-tolerant strains, stabilising agents like thiols or peptides, and buffering or surface coating methods to maintain nanoparticle integrity and reduce aggregation [56]. Further research is needed to optimise these approaches for robust, scalable nanoparticle biosynthesis.

## 3. Mechanisms of AgNPs Biosynthesis

The mechanism underlying the biosynthesis of AgNPs, particularly by bacteria, is generally more complex than that of chemical synthesis processes. Nonetheless, efforts have been made in this area, and several possible mechanistic models and pathways have been proposed.

### 3.1. Adaptive Nano Biogenesis

The production of AgNPs by bacteria has become a biologically and ecologically significant phenomenon, commonly referred to as nano biogenesis [57]. This process is not merely incidental but represents a vital survival mechanism in response to the inherent cytotoxicity of silver ions (Ag^+^). Examining the molecular interactions between silver ions and essential cellular components is deemed necessary to comprehend biochemical and physiological microbial behaviour [58,59].

**Figure 1:**
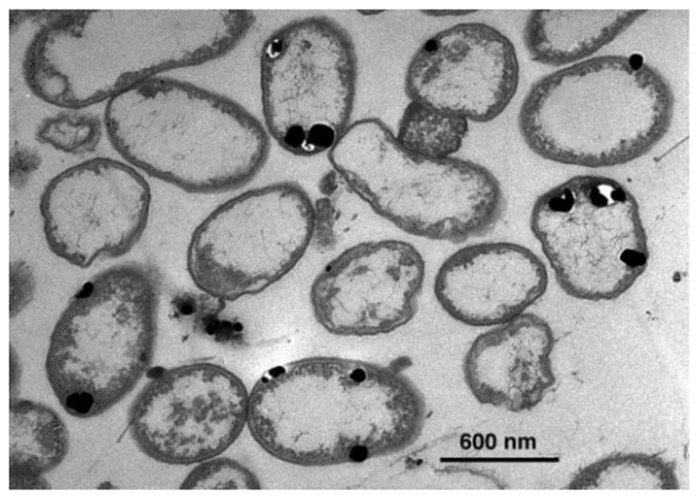
Transmission electron micrograph (TEM) of a thin section of *P. stutzeri* AG259 cells. Large crystalline Ag^0^ and Ag_2_ S particles are deposited between the cell wall and the plasma membrane [43]. (Copyright (1999) National Academy of Sciences, U.S.A.).

Silver ions are highly reactive and because of their tremendous affinity for biologically important macromolecules, especially those with electron-rich functional groups like thiols (-SH) and amines (-NH_2_), they exhibit potent antimicrobial activity. When exposed, Ag^+^ quickly displaces hydrogen atoms from sulfhydryl groups on microbial surfaces to produce stable silver-sulphur (S-Ag) bonds [60]. This interaction is seen in Figure 1, which illustrates the deposition of silver-based nanoparticles between the bacterial cell wall and membrane. The image highlights the direct involvement of silver ions in altering membrane-associated macromolecular structures [43].

This binding interferes with the structure and function of proteins by targeting the thiol side chains of cysteine residues, resulting in the cleavage of disulfide bonds, destabilisation of tertiary protein structures, and enzymatic inactivation. Since many enzymes rely on intact thiol groups for catalytic activity, this interaction has a significant impact on cell viability. In addition to protein disruption, Ag^+^ infiltrates the bacterial membrane, which is already compromised by the collapse of the proton motive force and respiratory inhibition and subsequently reaches the cytoplasm. There, it interferes with nucleic acid metabolism by creating coordination complexes with nitrogenous bases, especially guanine at the N7 atom. This interaction disrupts genomic integrity, prevents transcription and DNA replication and promoting pyrimidine dimerization [43,61]. Collectively, these disruptions impair critical biosynthetic pathways, including DNA, RNA, protein, and peptidoglycan synthesis, resulting in widespread metabolic failure.

In certain cases, Ag-induced oxidative stress and the buildup of reactive oxygen species (ROS) cause bacteria to display apoptosis-like reactions like DNA fragmentation and cytoplasmic condensation, which are characteristics of programmed cell death. This is likely due to the inhibition of redox-active enzymes like NADPH dehydrogenase II. With these multifactorial toxic effects, the bacteria’s capacity to produce AgNPs can be interpreted as an evolved detoxification strategy. By enzymatically reducing highly reactive Ag^+^ to its inert elemental form (Ag^0^), bacteria can effectively diminish the ionic bioavailability of silver and mitigate its cytotoxic potential [62,63,64]. This redox transformation is facilitated by membrane-bound or cytoplasmic reductases and electron shuttles that mediate electron transfer, although the exact biochemical mechanisms remain under investigation. The nanoparticles thus formed typically within extracellular surface or in the periplasmic space, are more stable, less reactive, and are incapable of inducing much biological harm as their ionic precursors.

In essence, the production of nanoparticles allows bacteria to sequester the resultant metallic silver in a physiologically inert form and neutralise toxic silver ions by bio-reduction. This technique not only promotes microbial survival in metal-rich settings, but it also demonstrates the remarkable adaptation capacities of living cells under chemical stress [65,66].

Ultimately, this process likely evolved as a survival strategy, granting bacteria a selective advantage and in doing so, bacteria not only mitigate silver’s antimicrobial effects but also generate nanoparticles through a biologically regulated bottom-up assembly process, using endogenous, non-toxic reducing agents in place of harsh chemicals. Although the broader ecological and technological implications of this microbial behaviour, particularly for green nanomaterial synthesis, are well recognised, the precise molecular pathways underlying AgNP biosynthesis remain poorly defined. Two principal mechanistic models have been proposed, as detailed below.

### 3.2. Development of Bacterial Resistance by Effluxing

One of the earliest and most well-characterised bacterial defence mechanisms against heavy metals toxicity involves a sophisticated efflux system [57,67,68,69]. These systems are membrane-bound transport proteins that recognise and actively extrude a wide range of toxic compounds including metal ions such as Ag^+^, Ag^2+^, Cu^2+^, Co^2+^, Zn^2+^, Cd^2+^, and Ni^2+^ from the intracellular environment, thereby maintaining intracellular homeostasis. In Gram-negative bacteria, efflux pumps contribute to both cytoplasmic and periplasmic detoxification, preventing metal ion accumulation that could otherwise re-enter the cytoplasm [70].

Depending on their energy source, efflux systems are classified as primary transporters, which utilise ATP hydrolysis, and secondary pumps, which rely on the proton motive force or ion gradients. When silver ions enter a bacterial cell, specific proteins recognise and initiate their active removal to prevent toxicity. Central to this process is the Sil operon, which encodes a suite of proteins, including the P-type ATPase (SilP) and the efflux transporter complex (SilCBA). Together, these components facilitate the expulsion of silver ions from the cytoplasm to the extracellular environment.

**Figure 2:**
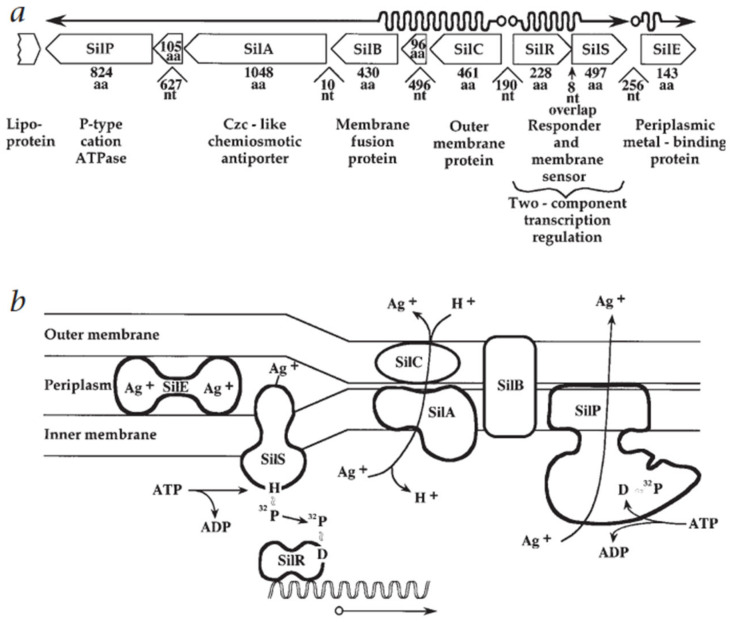
Genetic layout and functional roles of silver resistance determinants. (**a**) Schematic representation of gene organisation and transcription in the silver resistance operon. Promoter regions and transcription start sites are indicated by open circles; mRNA synthesis is depicted as wavy lines, and gene orientations are shown as arrows. Intergenic nucleotide (nt) distances and gene product sizes (in amino acids, aa) are noted. (**b**) Predicted protein functions based on homology, involved in Ag^+^ detection, transport, and detoxification [71]. (Adapted with permission from Springer Nature.).

Figure 2 illustrates this silver resistance mechanism model, showing the coordinated action of Sil system components. As depicted, SilP actively transports Ag^+^ ions from the cytoplasm to the periplasm, where SilF and SilE bind and sequester the ions. The SilCBA tripartite complex, which spans both the inner and outer membranes, completes the efflux by exporting the ions from the periplasm to the extracellular space. This multi-component pathway provides a rapid and effective response to acute silver stress [71,72].

This mechanism is functionally analogous to the Cus system, which performs a similar role in copper ion (Cu^+^) detoxification. In addition to SilP and SilCBA, the Sil system includes Ag^+^-binding chaperones such as SilF, and uniquely, SilE is a highly specific periplasmic silver-sequestering protein capable of binding up to ten Ag^+^ ions per peptide. These proteins act synergistically to sequester and expel toxic silver ions, providing the bacterium with short-term protection in silver-rich environments. Interestingly, homologs of Sil genes have been identified in bacteria capable of producing AgNPs, suggesting a potential connection between metal resistance and biogenic nanoparticle synthesis [72,73,74].

Overall, there are six major families of efflux pumps characterised in bacteria such as the ATP-binding cassette (ABC) superfamily, major facilitator superfamily (MFS), multidrug and toxic compound extrusion (MATE) family, resistance-nodulation-cell division (RND) family, small multidrug resistance (SMR) family, and the proteobacterial antimicrobial compound efflux (PACE) family. Among these, the RND family is most prevalent in Gram-negative bacteria, where it spans both the inner and outer membranes, plays a major role in the efflux of heavy metal ions like Ag^+^ and Cu^+^ as well as antibiotics. These systems are highly versatile, exhibiting substrate redundancy whereby a single pump can extrude a wide array of structurally diverse compounds, and conversely a single compound may be expelled by multiple pump types. This functional overlap not only enhances the bacterial capacity to survive diverse environmental stresses but also complicates therapeutic targeting [75].

While efflux systems are critical for mitigating acute silver toxicity, they are insufficient for ensuring long-term survival in persistently toxic environments. This limitation highlights the need for more durable detoxification mechanisms, such as the enzymatic reduction of Ag^+^ to elemental silver (Ag^0^), followed by the formation of AgNPs, which are chemically inert and significantly less toxic to the cell. To understand how bacteria shift from short-term efflux-based responses to more stable, long-term resistance strategies, the second prominent mechanism of silver resistance biogenic nanoparticle synthesis comes into focus [76]. Beyond efflux, a more enduring strategy to mitigate silver ion toxicity relies on enzymatic reduction of silver ions to its elemental form. Unlike silver ions, elemental silver is chemically inert and considerably less toxic, existing as a critical survival tool under chronic silver exposure. Once reduced, Ag^0^ atoms undergo nucleation and self-assembly, ultimately forming AgNPs via a biologically mediated bottom-up synthesis pathway, in contrast to conventional seed mediated chemical methods that rely on harsh reducing agents like sodium borohydride.

The mechanism of this biochemical reduction is further explained using two main hypotheses [77]. The first hypothesis suggests that simple biochemical agents, particularly aldehyde groups found in reducing sugars, serve as electron donors that facilitate the reduction of silver ions to elemental silver. While this model is supported by observations in certain *Lactobacillus* species known to synthesise AgNPs, it falls short in explaining the species-specific nature of AgNP formation, as most bacteria produce reducing sugars, yet only a limited number are capable of generating nanoparticles [55]. This limitation has given rise to a more plausible hypothesis centred on enzyme-mediated reduction. According to this hypothesis, specialised reductase enzymes likely part of the bacterial oxidative stress response transfer electrons to silver ions, catalysing their reduction in a regulated manner. This enzymatic hypothesis explained in depth in the following topic, aligns with emerging biochemical and genetic evidence and provides a more robust framework for understanding microbial silver resistance.

### 3.3. Intracellular and Extracellular Biosynthetic Pathways to AgNPs

The biosynthesis of nanoparticles is a complex process that remains under active investigation, as different microorganisms interact with metal ions in distinct ways. Broadly, two principal pathways have been identified: intracellular and extracellular synthesis [78,79]. In the intracellular pathway, metal ions first electrostatically bind to the negatively charged microbial cell walls, after which they are reduced to nanoparticles by enzymes located within or near the cell membrane. These newly formed nanoparticles may then diffuse out of the cell. Different organisms exhibit variations in this mechanism [80]. For instance, *Verticillium* species follow a sequential process involving metal ion trapping, bio-reduction, and capping [28]. While in *Lactobacillus* species, metal ion clusters nucleate and migrate through the cell wall, while in actinomycetes, the reduction takes place both at the mycelial surface and along the cytoplasmic membrane [55].

The intracellular reduction of metal ions into nanoparticles is also considered a microbial survival strategy to detoxify harmful ions. Typically, bacterial cells are incubated in a silver salt-containing medium under appropriate conditions, though resuspending the cells in sterile distilled water prior to silver exposure can reduce interference from media components. Notably, *Pseudomonas stutzeri* produces triangular and hexagonal nanoparticles within the periplasmic space [43], and *Acinetobacter* has shown biocompatibility with its own biosynthesized nanoparticles though its population declines upon exposure to silver salts. This selective compatibility is likely due to the presence of bacterial biomolecules that coat and stabilise the nanoparticles, making them non-toxic to the host [81].

**Figure 3:**
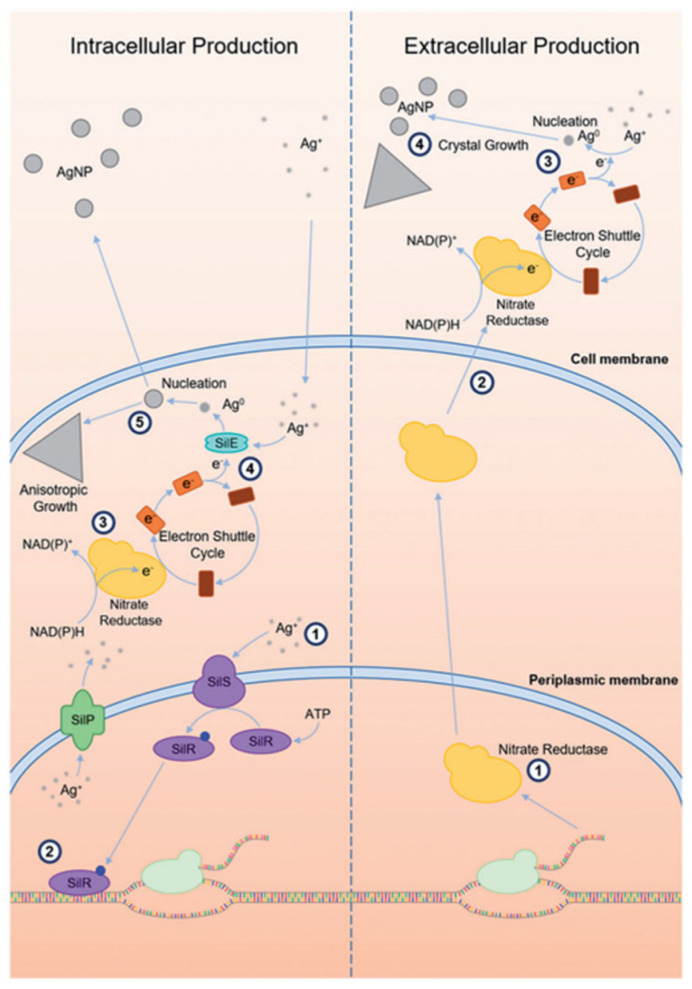
Schematic overview of intracellular and extracellular biosynthesis pathways of AgNPs in microorganisms. The intracellular route involves ion uptake, enzymatic reduction by nitrate reductase, and nanoparticle formation within the cell. In contrast, the extracellular route relies on secreted enzymes and electron shuttles to facilitate Ag^+^ reduction in the surrounding medium [21]. (Adapted with permission from Taylor and Francis.).

Furthermore, the size of intracellular AgNPs is influenced by the composition of the culture medium [82]. Despite its biological significance, intracellular synthesis is generally less preferred due to the additional steps required to recover the nanoparticles from within the cells. This process is depicted in Figure 3, exhibiting the two primary pathways, intracellular and extracellular production of microbial silver nanoparticle synthesis [21].

In the intracellular pathway (left), silver ions enter microbial cells and get reduced to elemental silver via intracellular enzymes such as nitrate reductase, often assisted by electron shuttle molecules. These reduced silver atoms then nucleate and grow into nanoparticles within the cell. In contrast, the extracellular route (right) involves the secretion of reductive enzymes and electron shuttles into the surrounding medium, allowing Ag^+^ ions to be reduced and facilitating nanoparticle formation externally [83]. In some organisms, such as *Morganella* species, periplasmic silver-binding proteins like SilE further enhance this process by directing Ag^+^ ions to the enzymatic reduction sites [84]. Due to its streamlined workflow and reduced downstream processing, this extracellular approach offers a more practical alternative for nanoparticle production.

Given this advantage, the extracellular synthesis pathway has garnered greater interest in its operational simplicity, efficiency and ease of nanoparticle recovery from the surrounding medium. At the core of this extracellular mechanism lies the activity of specific enzyme NAD(P)H-dependent nitrate reductase which has emerged as a key protagonist in driving the reduction of silver ions in various bacterial and fungal systems. This enzyme, typically classified within the dimethylsulfoxide reductase (DMSOR) family, facilitates the reduction of nitrate (NO_3_^−^) to nitrite (NO_2_^−^) through electron transfer donated from NADH or NADPH. These electrons travel through a chain of iron-sulphur clusters to a molybdenum (Mo) active site, where nitrate usually undergoes reduction. However, in the presence of silver ions, the enzymatic electron flow is redirected. Instead of reducing the nitrate, the electrons are passed on to Ag^+^ ions, resulting in their conversion into elemental silver atoms. These silver atoms serve as the nucleation units, which are the fundamental building blocks for the formation of AgNPs [85].

In numerous cases, this biochemical process is mediated by electron shuttle molecules such as 4-hydroxyquinoline, which facilitate indirect electron transfer from the enzyme to the silver ions. This mechanism is notably observed in the fungus Fusarium oxysporum, where nitrate reductase alone proved insufficient for efficient silver nanoparticle synthesis. Initially, NADPH donates electrons to a nitrate reductase enzyme, which typically reduces nitrate (NO_3_^−^) to nitrite (NO_2_^−^). However, in the presence of an electron shuttle such as 4-hydroxyquinoline, these electrons are instead relayed to silver ions (Ag^+^), effectively reducing them to elemental silver (Ag^0^) and enabling the formation of AgNPs [83,86].

This intricate biosynthetic mechanism is also prominently observed in microbial systems beyond fungi, where enzymatic processes involving a cascade of biochemical components drive the reduction of silver ions to elemental silver, resulting in nanoparticle formation. In particular, bacteria such as *Pseudomonas aeruginosa* and *Bacillus subtilis* have demonstrated the ability to synthesise NPs using only their cell-free extracts (CFEs), affirming that enzymatic constituents alone independent are sufficient to catalyse silver reduction [87].

This enzymatic activity is further exemplified in psychrophilic bacterial species. Recent studies have identified *Arthrobacter kerguelensis*, *A. gangotriensis*, *Pseudomonas antarctica*, *P. proteolytica*, and *P. meridiana*, alongside mesophilic strains such as *Bacillus indicus* and *B. cecembensis*, are capable of mediating extracellular AgNP biosynthesis. Notably, *Arthrobacter*, a genus not previously associated with nanoparticle synthesis, demonstrated efficient AgNP formation through its culture supernatants. Supernatants from *A. kerguelensis* and *P. antarctica* initiated AgNP synthesis within two hours considerably faster than most documented microbial systems.

However, nanoparticle stability varied, with *A. kerguelensis* producing more stable particles, whereas *P. antarctica* showed signs of aggregation within six hours, likely due to differences in the composition or concentration of stabilising agents. Interestingly, nutrient broth alone could mediate Ag^+^ reduction, although with markedly lower efficiency. Among its components, peptone exhibited the highest reducing capacity, followed by beef extract and NaCl. Nevertheless, the superior performance of bacterial supernatants in both synthesis rate and nanoparticle stability suggests the involvement of specific secreted biomolecules potentially proteinaceous capping agents or enzyme-derived cofactors that are essential for controlled nucleation, growth, and long-term stabilisation of biogenic AgNPs [34,41].

Other microorganisms, including *Thermomonospora* sp. and the cyanobacterium *Plectonema boryanum*, also perform extracellular nanoparticle synthesis. Notably, *Plectonema* exhibits temperature-dependent behaviour: at 60 °C, AgNPs form on the cell surface, while at 100 °C, cells become fully encrusted with AgNPs. The size of nanoparticles ranges from 1 to 40 nm inside cells to 1 to 200 nm outside, highlighting the significant role of environmental factors on extracellular nanoparticle synthesis [88]. While in silver-resistant *Escherichia coli* strain 116 AR, the biosynthesis of AgNPs has been closely linked to the activity of NapC, a tetra-heme c-type cytochrome subunit of the periplasmic nitrate reductase complex. NapC facilitates electron transfer from the cytoplasmic membrane’s quinol pool to the periplasm, where Ag^+^ ions undergo enzymatic reduction. Functional studies have confirmed the critical role of NapC; its genetic deletion significantly impaired AgNP production, whereas reintroduction of the gene partially restored nanoparticle synthesis [89].

In a parallel mechanism, *Morganella* species deploy the SilE protein, a periplasmic silver-binding factor, to chelate Ag^+^ and direct it toward the cellular reduction apparatus. This protein-mediated transport and presentation of silver ions enhances efficiency and contributes to the uniform morphology of the resulting nanoparticles [84]. Furthermore, dissimilatory metal-reducing bacteria such as *Shewanella oneidensis* MR-1 utilise complex networks of periplasmic and outer membrane c-type cytochromes originally adapted for anaerobic respiration to catalyse extracellular metal ion reductions [90]. Despite their potential, the application of these systems in silver nanoparticle biosynthesis remains underexplored, representing a promising avenue for future research.

The enzyme-mediated biosynthesis of AgNPs in microbial systems is a multifaceted process involving NAD(P)H as the primary electron donor, enzymatic catalysts such as nitrate reductase or c-type cytochromes, and silver-binding agents like SilE that aid in both the reduction and stabilisation of Ag^+^ ions [25,70,89]. Overall, while both mechanisms reflect nature’s remarkable adaptability, the extracellular approach offers a more practical, controllable, and sustainable strategy for the large-scale production of biologically functional AgNPs. This biologically driven conversion of toxic silver ions into functional nanomaterials presents a green alternative to traditional chemical synthesis, often yielding nanoparticles with desirable and tuneable physicochemical properties. Nevertheless, key environmental and physiological parameters such as pH, temperature, incubation duration, aerobicity and the presence of extracellular biomolecules play critical roles in influencing nanoparticle characteristics, including size, morphology, and monodispersity. Understanding and optimising these variables is essential to harness the full potential of microbial systems in AgNP synthesis.

## 4. Strategic Design of NPs’ Size and Shape for Enhanced Performance

Incredible properties of nanoparticles strongly depend on the size and shape, their interactions with stabilisers and surrounding media as well as their method of synthesis. Therefore, controlled synthesis of nanoparticles is essential to achieve desirable performance. This is particularly relevant for drug delivery applications, as they can enhance solubility, stability, targeted delivery, circulation time, and reduce side effects, positioning them as strong alternatives to conventional drug formulations [91]. However, as outlined in Table 2, these benefits are accompanied by significant challenges, including high production costs, toxicity, lack of reproducibility, limited clinical translation, and formulation complexity.

**Figure 4:**
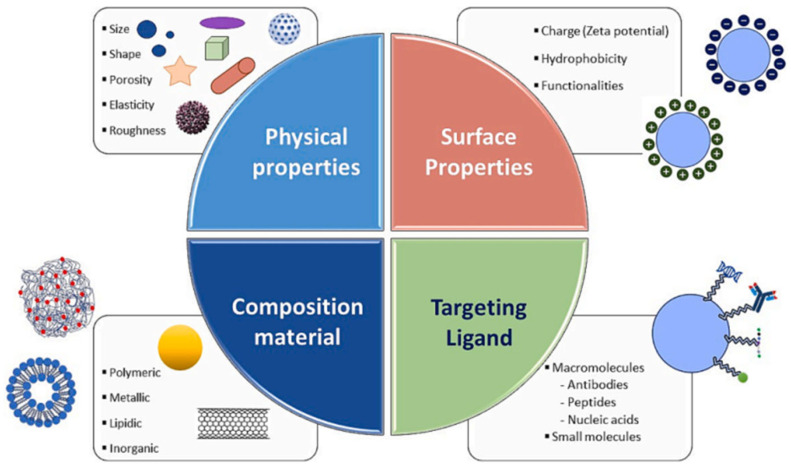
Tuneable physicochemical characteristics of nanostructures [92]. (Adapted with permission from Elsevier.).

While several nanomedicines, such as liposomes and polymeric nanoparticles, have received FDA approval, many others continue to face obstacles in clinical development due to factors including limited efficacy, undesirable side effects, suboptimal patient selection, and financial constraints [93]. These challenges highlight the critical need for rational and strategic formulation. In this context, tailoring the physicochemical properties of nanoparticles particularly their shape and size becomes essential for enhancing therapeutic performance. This section, therefore, focuses on how these parameters influence the in vitro and in vivo behaviour of nanostructures, with the aim of guiding the design of more effective and clinically translatable nano drug delivery platforms (Figure 4).

Nanoparticle size plays a critical role in drug delivery performance, influencing key in vivo parameters such as circulation time, biodistribution, cellular uptake, and tissue penetration. Generally, nanoparticles in the 2–200 nm range are considered optimal for intravenous applications, as they can evade rapid clearance by macrophages and leverage the enhanced permeability and retention (EPR) effect for tumour targeting [94]. Conversely, larger particles (>500 nm) are more readily phagocytosed and can trigger immune responses, beneficial for vaccine delivery or immune-related therapies but undesirable for systemic drug delivery.

Similarly, particle shape plays a crucial role in determining biological behaviour and therapeutic potential. While spherical nanoparticles are commonly used due to their ease of synthesis, non-spherical particles, such as rods, filaments, and disks, are gaining attention for their ability to mimic pathogens, evade the immune system, and more effectively traverse biological barriers. High-aspect-ratio particles have shown promising results in enhancing tumour homing and cellular interactions, particularly through the enhanced permeability and retention (EPR) effect, which favours particles in the 10–150 nm range for optimal penetration and retention. However, rod-shaped nanoparticles may exhibit slower internalisation than spherical ones, depending on their orientation relative to the cell membrane [92,95,96]. Therefore, fine-tuning nanoparticle size and shape not only affects internalisation efficiency but also governs biological fate, circulation time, and site-specific delivery, but also reasons the necessity of strategic design based on the intended therapeutic goal.

Advanced fabrication techniques such as PRINT (Particle Replication In Non-wetting Templates) [97], film-stretching, and self-assembly enable precise control over the size and shape of nanoparticles. This level of control allows for the design of tailor-made nanocarriers that align with specific therapeutic objectives, whether rapid delivery for acute treatments, sustained release for chronic conditions, or targeted delivery to tumours or intracellular organelles. For example, high-aspect-ratio particles engineered via PRINT have demonstrated superior tumour targeting and retention, while disk-shaped particles have shown enhanced adhesion to vascular walls, making them well-suited for cardiovascular applications [98]. These findings outline that there is no one-size-fits-all design; instead, the optimisation of nanoparticle size and shape must be aligned with the intended route of administration, target tissue or organ, drug properties, and therapeutic goals to maximise efficacy and minimise off-target effects.

### 4.1. Impact of AgNPs’ Shape and Crystallographic Facets on Antibacterial Activity

The global rise in antibiotic-resistant bacteria has intensified interest in exploring nanomaterials, particularly AgNPs, as synergistic antimicrobial agents [99]. Among the various physicochemical parameters influencing AgNP efficacy, particle morphology, especially shape, size, and crystallographic facets, plays a critical role in modulating their interactions with bacterial cells and the resulting bactericidal mechanisms [100,101].

A growing body of literature has demonstrated that the geometric anisotropy of AgNPs significantly influences their antibacterial performance. Specifically, triangular silver nanoplates or nanotriangles, characterised by their sharp vertex edges and high aspect ratios, consistently outperform spherical and rod-shaped nanoparticles in antimicrobial efficacy. The enhanced activity of nanotriangles is largely attributed to their high-energy {111} crystallographic facets and large surface area-to-volume ratio. These features allow effective adhesion to bacterial membranes and disrupt membrane integrity, especially in both Gram-positive (e.g., *Staphylococcus aureus*) and Gram-negative (e.g., *Escherichia coli*) strains which ultimately leads to cell lysis [102].

**Figure 5:**
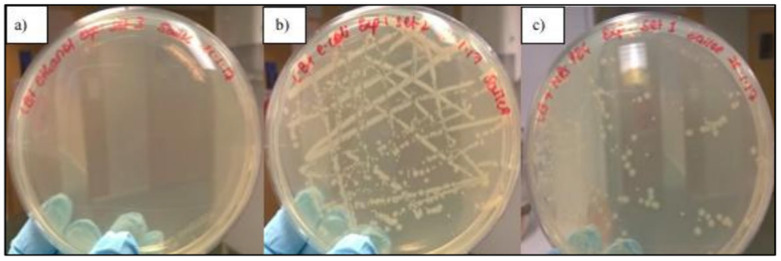
Inhibitory effect of PEGylated silver nanotriangles on *E. coli* after 24 h incubation on 1.5% LB agar. (**a**) Positive control (70% ethanol) showed no growth. (**b**) Negative control (untreated plate) exhibited dense colonies. (**c**) PEG-AgNT-treated plate showed significantly reduced colony formation. White dots indicate *E. coli* colonies. Plates supplemented with PEG AgNTs exhibited fewer colonies compared to the negative control [105]. (Courtesy of Sailee Shroff.).

Quantitative comparative analyses reveal a consistent hierarchy in antimicrobial activity: nanoplates > nanorods > spherical nanoparticles, based on inhibition zone size, kill time, and minimum inhibitory concentration (MIC) values. The superior antimicrobial efficacy of anisotropic AgNPs is further supported by comparative inhibition zone assays. For instance, polyvinylpyrrolidone (PVP)-capped silver nanoplates produced inhibition zones of 9 mm against *Staphylococcus aureus* and 4 mm against *Escherichia coli*, whereas spherical nanoparticles of equivalent stabilisation achieved only 2 mm and 1 mm, respectively, under identical conditions [103,104]. Complementing these findings, the biocidal activity of PEGylated silver nanotriangles (PEG AgNTs) was specifically evaluated against *E. coli* (DH5α) using both qualitative and quantitative methods. As shown in Figure 5, PEG-AgNT-treated agar plates exhibited a marked reduction in bacterial growth, with only 103 colonies observed after 24 h, compared to complete inhibition by 70% ethanol (positive control) and dense colony formation in the untreated control [105].

These findings highlight the importance of facet-rich, planar morphologies in enhancing nanoparticle contact with bacterial cell surfaces and exerting greater mechanical stress compared to their spherical or rod-shaped counterparts.

### 4.2. Crystallographic Facets and Surface Reactivity

As discussed above, the antimicrobial efficacy of AgNPs is significantly influenced by their exposed specific crystallographic facets governing surface reactivity and interaction with microbial cells. In particular, triangular and decahedral AgNPs predominantly expose high-atom-density {111} facets known for their superior surface energy and stability. These facets facilitate enhanced adhesion to bacterial membranes, resulting in membrane disruption, promote Ag^+^ ion release, and enable intracellular penetration, where they interfere with DNA, protein function, and other essential biomolecular pathways [102,106].

**Figure 6:**
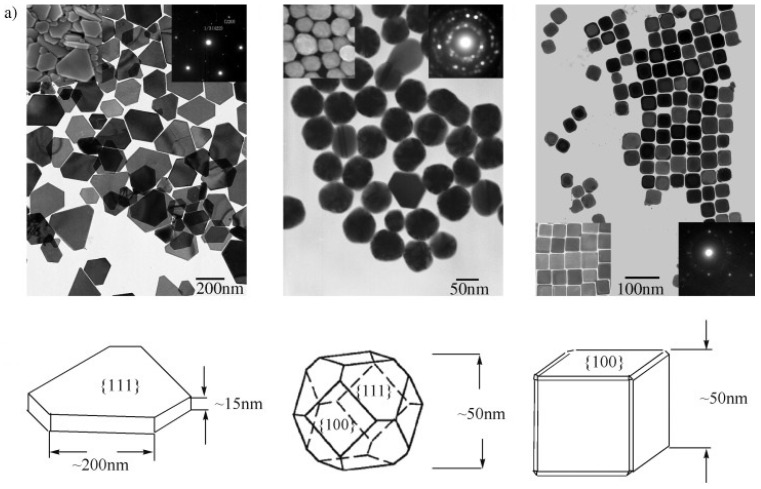

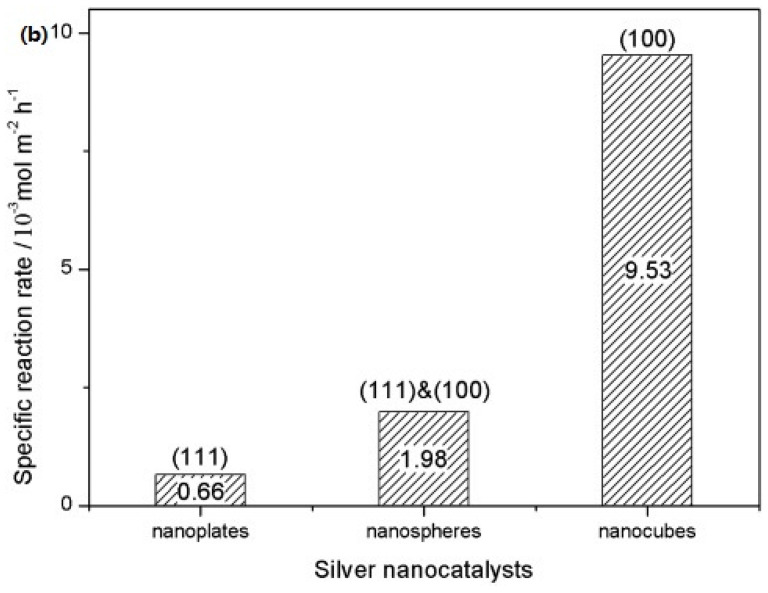
(**a**) TEM images of truncated triangular, near-spherical, and cubic Ag nanoparticles on Cu-TEM grids with corresponding SEM images and electron diffraction patterns. (**b**) Specific reaction rates for styrene conversion over shape-controlled Ag NPs after 3 h [108]. (Adapted with permission from John Wiley and Sons.).

In contrast, spherical AgNPs typically expose a mix of crystallographic facets having lower surface energy and reduced surface reactivity, correlating with reduced antimicrobial activity. Rod-shaped AgNPs have {100} facets along the sides and {111} facets at the termini, offering intermediate bactericidal potency. Structural characterisations like OPML-XRD consistently reveal that dominance of {111} facets correlate strongly with enhanced antibacterial behaviour, particularly in Gram-negative bacteria where the thinner peptidoglycan layer of 7–8 nm facilitates deeper nanoparticle infiltration and membrane disruption [106,107]. Supporting these findings, a study by Xu et al. examined the shape-dependent catalytic performance of AgNPs in the oxidation of styrene. Their results (Figure 6) showed that nanocubes exhibited a reaction rate 14 times higher than triangular nanoplates and 4 times greater than semi-spherical particles, emphasising the critical role of facet orientation in determining nanoparticle reactivity [108].

### 4.3. Size-Dependent Antibacterial Activity and Surface Area Effects

In addition to morphology and crystallographic orientation, nanoparticle size plays a crucial role in determining antibacterial efficacy. Smaller AgNPs, particularly those with diameters below 10 nm, exhibit superior antibacterial activity, primarily due to their higher surface area-to-volume ratio. This increased surface availability enhances interactions with bacterial membranes and promotes a higher rate of Ag^+^ ion dissolution. Empirical data show that 2–5 nm spherical AgNPs inhibit bacterial growth more effectively than larger penta- and hexagonal particles measuring approximately 50–100 nm, despite the latter possessing structurally active edges.

Notably, even among similarly sized nanoparticles, geometry continues to influence performance. Penta- and hexagonal-shaped particles, owing to their multiple reactive edges, outperformed larger spherical particles by 15–18% in inhibition assays. This enhanced performance is attributed to the greater number of high-energy edge sites and facet terminations. The synergistic combination of small size and facet-rich geometry optimises both physicochemical reactivity and mechanical disruption, thereby maximising antimicrobial potency [45,109].

### 4.4. Mechanistic Insights into Bactericidal Activity

The mechanistic basis for the superior antimicrobial performance of certain AgNP morphologies lies in their distinct physicochemical interactions with bacterial cell membranes and intracellular targets. High-aspect-ratio and sharp-edged structures, such as triangular silver nanoplates and decahedral AgNPs, induce physical puncturing and deformation of bacterial membranes [110]. This mechanical disruption is further complemented by elevated Ag^+^ ion release, which is modulated by both facet orientation and particle size. The released silver ions exert cytotoxic effects through multiple pathways, including the generation of reactive oxygen species (ROS), interference with enzyme function via thiol binding, and the induction of genotoxic stress through direct DNA interaction, all culminating in bacterial cell death [111,112]. Furthermore, synergistic studies have shown that facet-optimised AgNPs can restore the activity of conventional antibiotics such as ampicillin and penicillin against multidrug-resistant bacterial strains. This suggests that morphology-engineered AgNPs not only exhibit intrinsic bactericidal activity but also function as adjuvants in combination therapies aimed at overcoming antibiotic resistance [113].

Although significant progress has been made in enhancing the antibacterial efficacy of AgNPs through morphological control, future research is required to prioritise targeted facet engineering, refined nanoscale design, and synergistic integration with conventional antibiotics to effectively address the growing threat of multidrug-resistant pathogens [114]. Beyond antimicrobial applications, ongoing investigations should expand to explore the broader biomedical potential of AgNPs. Fine-tuning their morphology and surface chemistry to enable multimodal functionality could pave the way for their use in drug delivery platforms, molecular imaging, tissue engineering, and cancer therapies.

## 5. Biomedical Applications of Biogenically Synthesised AgNPs

Nanotechnology has profoundly influenced biomedical sciences, with AgNPs emerging as one of the most extensively studied and applied nanomaterials. Their distinctive physicochemical properties including ultra-small size, high surface area-to-volume ratio, and adjustable shape and surface chemistry make AgNPs highly versatile for diverse biomedical applications. AgNPs are widely integrated into antimicrobial coatings for medical devices such as catheters, wound dressings, and implants, leveraging their strong bactericidal and fungicidal effects. Beyond antimicrobial use, AgNPs have demonstrated promising potential in targeted drug delivery systems, cancer therapy, biosensing, imaging, and regenerative medicine. For example, smaller-sized AgNPs with controlled morphology have been shown to enhance cellular uptake and therapeutic efficacy in drug delivery, while also posing less risk of toxicity when carefully engineered [115].

Furthermore, concerns over the cytotoxicity and environmental impact of chemically synthesised AgNPs have prompted a shift towards biogenic synthesis methods. Biogenic or green synthesis of AgNPs employs biological systems such as plant extracts, fungi, or bacteria, offering an eco-friendly and safer alternative to conventional chemical and physical synthesis. This approach not only reduces the use of hazardous chemicals but also incorporates naturally occurring biomolecules as capping and stabilising agents, which can improve biocompatibility and reduce the release of toxic silver ions. Importantly, plant-mediated synthesis methods have been reported to produce AgNPs with tailored size and shape, directly influencing their biomedical performance. For example, biogenically synthesised AgNPs have shown reduced cytotoxicity in human cell lines while maintaining effective antimicrobial activity, making them particularly attractive for applications in wound healing, antibacterial coatings, and tissue engineering [116]. As such, biogenic AgNPs present a promising and safer avenue for expanding nanotechnology’s role in biomedical applications, aligning material innovation with health and environmental safety considerations.

### 5.1. Synergistic Effects on Antibiotics

One of the most promising applications of AgNPs in antimicrobial therapy lies in their ability to synergise with conventional antibiotics, particularly against multidrug-resistant (MDR) bacteria. AgNPs biosynthesized using the fungus *Trichoderma viride*, have displayed to boost the antimicrobial efficacy of antibiotics including ampicillin, kanamycin, erythromycin, and chloramphenicol [113]. Among these, the most pronounced synergistic effect was observed with ampicillin, offered a substantial increase in the inhibition zone when co-administered with AgNPs. This enhancement is visually represented in Table 3, which compares inhibition zone diameters for different antibiotics administered alone and in combination with various AgNP morphologies [117].

This synergism is profoundly size and shape dependent. Notably, co-administration with spherical AgNPs in the 2–5 nm range were especially effective in enhancing the action of β-lactam antibiotics like penicillin and ampicillin particularly against resistant strains. In contrast pentagonal and hexagonal AgNPs with distinct edges further enhanced inhibition activity, with up to a 45% increase in some sizes compared to antibiotic treatment alone. Notably, this shape- and size-dependent interaction was more prominent with antibiotics to which bacteria had already developed resistance, emphasising the potential of nanoparticle engineering in combating antimicrobial resistance [117]. Therefore, rational and precise design of these parameters is essential for creating advanced antimicrobial agents that can effectively tackle the rising threat of antibiotic-resistant infections worldwide.

### 5.2. Anticancer Applications

Beyond antimicrobial properties, AgNPs have shown notable potential in cancer therapy. Sheikpranbabu et al. demonstrated that biogenically synthesised AgNPs can effectively inhibit angiogenesis in bovine retinal endothelial cells by disrupting VEGF signalling, activating caspase-3, and inducing apoptosis. This anti-angiogenic action positions AgNPs as potential therapeutic agents for limiting tumour vascularisation [118]. Further supporting their anticancer efficacy AgNPs sized between 5 and 60 nm have exhibited concentration-dependent cytotoxicity in cancer cells such as breast cancer and colon cancer. Notably, green-synthesised triangular and hexagonal AgNPs outperform spherical ones due to their enhanced surface reactivity and ability to generate reactive oxygen species (ROS), which contributes to DNA damage and initiate programmed cell death [119]. This structural advantage not only promotes more effective induction of apoptosis in cancer cells but also improves the overall therapeutic potential of AgNPs in targeted cancer treatment strategies.

Recent studies have further elucidated the mechanisms and broadened the scope of biogenically synthesised AgNPs in oncology. For instance, green-synthesised AgNPs using plant extracts, such as those derived from *Azadirachta indica* (neem), have shown promising results against lung cancer cell lines (e.g., A549) [120]. Additionally, biogenically synthesised AgNPs were shown to overcome multidrug resistance in cancer. These nanoparticles, crafted using fungal extracts like *Aspergillus niger*, disrupted efflux pumps in resistant breast cancer cells (MCF-7/ADR), enhancing the intracellular accumulation of chemotherapeutic agents like doxorubicin [121]. The biocompatibility of biogenically synthesised AgNPs is a key advantage, as capping agents from natural sources (e.g., polyphenols, flavonoids) reduce toxicity to normal cells, such as fibroblasts, compared to chemically synthesised counterparts [122].

Moreover, recent advances have explored AgNPs in combination therapies. Studies also demonstrated that biogenically synthesised AgNPs synergised with photodynamic therapy to target cancerous cells by amplifying ROS production and triggering apoptosis under light irradiation [123]. Size and shape continue to play critical roles, with smaller AgNPs (5–20 nm) penetrating tumour tissues more effectively where biogenically synthesised rod-shaped AgNPs exhibited superior uptake and cytotoxicity in pancreatic cancer cells compared to larger spherical particles [124]. Collectively, these studies highlight the expanding role of biogenically synthesised AgNPs as potent, eco-friendly tools in cancer therapy. However, challenges remain, including the optimisation of nanoparticle stability and the need for selective targeting to minimise off-target effects on healthy tissues [20].

### 5.3. Roles in Targeted Drug Delivery

AgNPs have also attracted significant interest as drug carriers due to their ability to be easily functionalised with therapeutic agents, targeting ligands, or polymers, allowing for tailored delivery to specific cell types or disease sites. Again, their size-dependent behaviour plays a critical role in determining the pharmacokinetics and biodistribution of AgNPs. AgNPs smaller than 50 nm can penetrate biological barriers effectively, accumulate in tumour tissues via the enhanced permeability and retention (EPR) effect, and are readily internalised via endocytosis. Studies reported that AgNPs sized 10–20 nm exhibited significantly higher uptake in MCF-7 breast cancer cells compared to larger counterparts, enhancing cytotoxic drug delivery.

However, rapid clearance from the body remains a concern for ultra-small particles, which can limit their circulation time and reduce therapeutic efficacy [119]. To address this, surface modifications such as polyethylene glycol (PEG) coating are generally employed to increase systemic retention and reduce immunogenicity, as demonstrated in a study by Wei et al. [125].

The shape of AgNPs also affects cellular uptake and drug release dynamics. While spherical AgNPs ensure uniform distribution and predictable cellular behaviour, other anisotropies such as rods, prisms, and triangular plates provide larger surface areas and reactive facets. These structural features enable more selective interactions with cellular membranes and allow for stronger attachment of targeting ligands or therapeutic molecules. Supporting this, another study demonstrated that rod-shaped AgNPs achieved significantly higher uptake and cytotoxicity in HeLa cells compared to spherical particles, attributing this to their superior membrane interaction and internalisation efficiency.

Moreover, the geometry of AgNPs also influences intracellular trafficking and drug release kinetics. Anisotropic shapes can enhance endosomal escape and promote sustained intracellular release, thereby improving therapeutic outcomes [92,126,127]. Overall, both size and shape are critical design parameters in optimising AgNP-based drug delivery systems. Through careful tailoring of morphology and surface modifications, these carriers can achieve higher drug loading, targeted delivery, and controlled release supporting more effective and personalised treatments for cancer and infectious diseases.

### 5.4. Next-Generation Biomedical Applications Enabled by AgNPs

The future of smart medical applications using AgNPs is poised for transformative advancements through the integration of cutting-edge technologies and biological insights. Smart systems such as pH-responsive hydrogels and nanofibers already demonstrate the ability to release AgNPs in response to environmental cues like pH shifts or bacterial presence, enabling targeted antimicrobial action while minimising toxicity. In particular, pH-responsive hydrogels are engineered to remain inert under normal physiological conditions but trigger the controlled release of silver ions when the local pH becomes alkaline. This is a change typically associated with bacterial infections. It ensures localised antimicrobial activity at infection sites, reducing unnecessary silver exposure and protecting healthy tissue. These hydrogels often utilise polymers that swell, degrade, or alter their network structure in response to pH variations, enabling precise spatial and temporal control over AgNP delivery. The result is a significant improvement in bacterial eradication efficiency, particularly against drug-resistant strains, while preserving mammalian cell viability [128].

In parallel, lab-on-a-chip (LOC) technologies incorporating AgNPs are emerging as highly effective tools for real-time, point-of-care diagnostics. These microfluidic platforms exploit the unique optical, catalytic, and antimicrobial properties of AgNPs for the rapid and sensitive detection of infectious agents, resistance genes, and disease biomarkers. Their miniaturised and portable design allows for low-cost, high-throughput analysis using minimal sample volumes, making them ideal for clinical settings, field use, and low-resource environments. AgNP-based LOC systems often employ biosensors and surface-enhanced Raman scattering (SERS) technologies to provide ultra-sensitive detection and real-time feedback, enabling timely and accurate therapeutic interventions [129].

AgNPs also show promise in antiviral applications, where they can disrupt multiple stages of the viral life cycle, including attachment, entry, and replication. This antiviral activity is largely attributed to their nanoscale size and surface morphology, which enable strong interactions with viral envelope proteins, altering their binding affinity and inhibiting infectivity. In photodynamic therapy (PDT), AgNPs serve to amplify the generation of reactive oxygen species (ROS), a mechanism that is enhanced by shape-dependent light absorption. Specific geometries such as truncated triangles or pentagons optimise this light-mediated therapeutic effect, improving outcomes in cancer and antimicrobial treatments.

Beyond therapeutic uses, AgNPs are being integrated into advanced medical textiles. For examples, silver-embedded fabrics synthesised via extracellular biosynthesis using fungi like *Fusarium oxysporum* exhibit potent antimicrobial effects, particularly against pathogens such as *Staphylococcus aureus*. These fabrics are well-suited for use in wound dressings, surgical garments, and hospital linens, contributing to infection control in clinical environments [83].

Despite their demonstrated utility, several aspects of AgNP biosynthesis remain only partially understood. For example, SDS-PAGE analyses of fungal culture supernatants from *Aureobasidium kerguelensis* and *Penicillium antarctica* have failed to identify the specific proteins responsible for nanoparticle formation, underscoring a significant knowledge gap. Addressing these gaps is essential for improving the reproducibility, scalability, and functional consistency of biogenic AgNPs, paving the way for their broader and more reliable application in medicine [130,131].

### 5.5. Toxicological Impacts of AgNPs on Human Health

AgNPs, widely utilised in medical devices, textiles, cosmetics, and food packaging due to their potent antimicrobial properties, have raised growing concerns regarding their potential cytotoxicity and genotoxicity in humans. These nanoparticles can enter the human body via inhalation, oral ingestion, dermal contact, and systemic circulation [132]. Following internalisation via cellular uptake mechanisms such as diffusion, endocytosis, and phagocytosis dependent on particle size and morphology, AgNPs release silver ions (Ag^+^), which are primarily responsible for inducing oxidative stress through the overproduction of reactive oxygen species (ROS). The resultant oxidative stress perturbs cellular redox balance, leading to mitochondrial damage, adenosine triphosphate (ATP) depletion, lipid peroxidation, protein carbonylation, DNA fragmentation, and ultimately apoptosis [126]. Experimental evidence highlights a strong size-dependent toxicity profile, wherein smaller nanoparticles less than 10 nm exhibit greater cytotoxicity due to their higher surface area-to-volume ratio and increased silver ion release [133,134]. For example, exposure of HepG2 human hepatoma cells to AgNPs at a concentration of 10 mg/L significantly compromised cell viability and elevated lactate dehydrogenase (LDH) membrane leakage, indicative of membrane integrity disruption.

Furthermore, AgNPs have demonstrated the ability to traverse physiological barriers such as the blood-brain and blood-testis barriers, leading to accumulation in organs such as the brain and testes, where they have been implicated in neurotoxicity and impaired spermatogenesis, respectively [115,135]. Protein–nanoparticle interactions represent another critical pathway of toxicity. AgNPs have been shown to bind with haemoglobin, altering its secondary structure and forming haemoglobin–AgNP complexes that may interfere with electron transport. Similar interactions with cytoskeletal proteins can compromise structural cellular components, leading to impaired intracellular transport and stability [136]. Environmental impacts are also profound where AgNPs disrupt soil microbial communities vital for nitrogen cycling and harm aquatic organisms by impairing osmoregulation in fish [115].

Notably, commercially available silver-based dressings have shown cytotoxicity in keratinocytes and fibroblasts, with toxicity levels correlating to silver release. Importantly, the method of AgNP synthesis plays a pivotal role in modulating their toxicological profile. Traditional chemical synthesis routes often involve toxic reagents and yield particles with high reactivity and poor biocompatibility. In contrast, green synthesis approaches employ biological materials such as plant extracts (e.g., *Acorus calamus*, *Gracilaria birdiae*) and microorganisms, which offer a more sustainable and safer alternative.

The phytochemicals present in these extracts such as polyphenols, flavonoids, proteins, and polysaccharides function as both reducing and capping agents, contributing to the stabilisation of nanoparticles and attenuating their ion release and ROS generation. This results in reduced cytotoxicity and enhanced biocompatibility [137]. Nevertheless, despite these promising findings, the review underscores the need for comprehensive in vivo studies to elucidate the long-term health impacts and ensure the safe integration of green-synthesised AgNPs in biomedical and industrial applications.

Furthermore, special attention must be given to the regulatory evaluation and safety profiling of pharmaceutical substances incorporating AgNPs. Rigorous preclinical and clinical assessments including pharmacokinetics, biodistribution, and toxicodynamics are essential to ensure patient safety and therapeutic efficacy. Recent studies have shown that surface modification and functionalisation of AgNPs with biocompatible polymers, such as polyethylene glycol (PEG), polyvinylpyrrolidone (PVP), and chitosan, can significantly reduce cytotoxicity, enhance colloidal stability, and improve pharmacological performance. These polymer coatings act as controlled-release barriers, modulating silver ion release rates, minimising oxidative stress, and reducing cellular damage while maintaining antimicrobial efficacy.

Moreover, functionalisation strategies involving targeting ligands or stimuli-responsive polymers are increasingly being explored to achieve site-specific delivery and reduce off-target effects, which is particularly relevant for pharmaceutical formulations [138,139]. It is also important to consider the aggregation behaviour of AgNPs in physiological environments, as polymer coatings have been shown to prevent unwanted agglomeration and protein corona formation, further enhancing biocompatibility and reducing unintended immune responses.

Therefore, establishing standardised protocols for physicochemical characterisation, quality control, and biocompatibility testing is imperative to support the responsible and safe application of AgNPs in pharmaceuticals. Such measures will not only help mitigate potential adverse effects but also facilitate regulatory approval and clinical translation, promoting the development of safe, effective, and sustainable silver nanoparticle-based therapeutics.

## 6. Concluding Remarks

The biosynthesis of AgNPs has emerged as a sustainable and eco-friendly alternative to conventional chemical synthesis methods. Leveraging the intrinsic metabolic and enzymatic capabilities of microorganisms, including bacteria, fungi, and microalgae, this biogenic approach enables the reduction of aqueous silver ions (Ag^+^) to elemental silver (Ag^0^) under mild conditions, providing useful nanomaterials for potential biomedical, environmental, and industrial applications.

This review consolidates current understanding of microbial biosynthesis pathways, including intracellular and extracellular routes, particularly those involving NAD(P)H-dependent nitrate reductase and electron transfer mechanisms. Extracellular synthesis, in particular, holds industrial promise due to easier scalability and nanoparticle recovery. In addition, studies in the literature have explored the use of natural humic substances as reducing and stabilising agents for the synthesis of AgNPs including the application of microwaves, offering an alternative route for the biosynthesis of AgNPs [31]. Notably, humic ligands complex assemblies of natural organic molecules with abundant electron-donating functional groups can effectively stabilise AgNPs while enhancing their bioavailability and antibacterial activity. Functional bionanomaterials, formed by ultradispersed AgNPs embedded in humic substance matrices, have shown not only direct antimicrobial effects but also the ability to penetrate bacterial biofilms and inhibit their formation, addressing challenges such as antibiotic resistance and biofilm-associated infections [138,139].

In biomedical contexts, biogenically synthesised AgNPs have demonstrated strong potential in diverse therapeutic applications as demonstrated in vivo and preclinical or clinical studies. These include antimicrobial treatments, cancer therapy, wound healing, and theranostics, enabled by their tenable surface properties and biocompatibility. Their size- and shape-dependent interactions with biological systems enhance cellular uptake, modulate immune responses, and allow for targeted delivery, reinforcing their versatility in clinical use.

However, significant challenges persist. Controlling nanoparticle size, shape, and crystallographic facets, especially the antimicrobial-enhancing {111} planes, remains technically difficult. Abiotic factors such as pH, temperature, oxygen levels, and media composition are critical but often underexplored determinants of synthesis efficiency and nanoparticle stability. The mechanistic understanding of enzymatic vs. non-enzymatic reduction remains incomplete, as does the genetic and regulatory framework that governs these biosynthetic pathways.

Furthermore, although microbial AgNP production reflects a sophisticated adaptive response to metal stress, transforming cytotoxic Ag^+^ into less harmful Ag^0^, the broader ecological and clinical implications demand closer scrutiny. Emerging concerns include the potential development of bacterial resistance, the nanoparticles’ interactions with host microbiota, and their long-term environmental impact.

## 7. Future Prospects and Industrial Challenges in the Biological Synthesis of AgNPs

The biological synthesis of AgNPs emerges as a sustainable and biocompatible alternative to conventional physical and chemical methods. Leveraging the natural reducing and stabilising capabilities of microorganisms (such as bacteria, fungi, and algae) and plant extracts, this approach enables the production of potent antimicrobial and therapeutic nanoparticles under mild and eco-friendly conditions. Looking ahead, the integration of multi-omics technologies including genomics, transcriptomics, proteomics, and metabolomics will provide crucial insights into the biosynthetic pathways and regulatory mechanisms governing nanoparticle formation. These advances will support the rational engineering of microbial strains with enhanced production efficiency and the development of species-specific protocols for generating application-tailored AgNPs [140].

The incorporation of machine learning and artificial intelligence (AI) into this field is also poised to revolutionise nanoparticle biosynthesis by enabling the prediction and optimisation of synthesis parameters such as pH, temperature, and precursor concentration leading to better control over particle size, morphology, and surface functionality. These tailored AgNPs can then be integrated into smart biomedical systems, such as drug delivery vehicles, biosensors, lab-on-a-chip platforms, and wound-healing dressings, to support next-generation diagnostic and therapeutic innovations.

Despite these encouraging prospects, the industrial-scale production of biologically synthesised AgNPs faces significant barriers. A major challenge is the incomplete mechanistic understanding of nanoparticle formation at the molecular level. For example, studies investigating protein involvement in nanoparticle synthesis using SDS-PAGE in organisms like *Fusarium oxysporum* and *Pseudomonas stutzeri* have struggled to identify the specific enzymes or peptides directly responsible for silver ion reduction and nanoparticle stabilisation, highlighting substantial gaps in our molecular understanding [83].

In addition, batch-to-batch variability, low production yield, and limited scalability of microbial or plant-based systems hinder reproducibility. Other constraints include poor control over nanoparticle uniformity, risks of biological contamination, and regulatory hurdles stemming from insufficient data on in vivo biosafety, toxicity, and long-term environmental impact.

To overcome these limitations, future research must focus on the genetic engineering of high yield microbial strains, the implementation of bioreactor-based production systems, and the development of standardised isolation and purification protocols. Moreover, establishing joint frameworks for clinical-grade nanoparticle production, including Good Manufacturing Practice (GMP) compliance and real-time quality control systems, will also support industrial scalability. Conducting robust in vivo evaluations and environmental assessments will be essential to validate the clinical safety and ecological sustainability of biologically synthesised AgNPs.

In conclusion, while challenges remain, the biological synthesis of AgNPs offers a transformative path towards environmentally responsible and functionally versatile nanomaterials. With ongoing technological integration and strategic problem solving, this field holds the potential to revolutionise biomedical applications through scalable, safe, and smart nanotechnology solutions.

## Figures and Tables

**Table 1:** Summary of the characteristics of the three biosynthetic methods for synthesising AgNPs.

Biosynthetic Method	Advantages	Disadvantages	Differences in Formed NPs	Ref.
**Plant-Mediated Synthesis**	- Simple and cost-effective - Eco-friendly, eliminates hazardous chemicals - Uses renewable resources (i.e., plant extracts) - High stability due to phytochemicals (flavonoids, terpenoids, phenolics) acting as reducing/capping agents - Scalable and reproducible	- Variability in extract composition due to plant species, geographical sources, and seasonal changes - Challenges in standardising extracts	- Size: 10–50 nm - Mostly spherical morphology - Potent antimicrobial and anticancer activity - Enhanced stability due to natural capping agents	[12,26,27]
**Microbial-Based Synthesis**	- Tailored properties via microbial metabolism - High yields with fungi (e.g., *Fusarium oxysporum*) - Natural capping agents from algae reduce aggregation - Antibacterial and anticancer effects	- Limited scalability due to controlled culture conditions (pH, temperature, nutrients) - Higher cost compared to plant-mediated synthesis - Optimisation needed for industrial-scale production	- Variable size and morphology depending on microorganism - Extracellular or intracellular synthesis - Enhanced antibacterial properties with optimised conditions	[13,21,28,29,30]
**Biomolecule-Based Synthesis**	- Precise control over particle size, morphology, and function - Enhanced biocompatibility (e.g., chitosan-stabilised AgNPs; humic ligands as reducing/stabilising agents) - Suitable for specific applications (e.g., drug delivery)	- Limited by availability and cost of purified biomolecules - Scalability challenges due to biomolecule production costs	- Highly controlled size and morphology - Stabilised by proteins or polysaccharides - Tailored for biomedical applications	[16,20,31,32]

**Table 2:** Advantages and challenges of nanosystems [92].

Advantage	Challenges
Advanced drug stability	Scale-up
Improved drug solubility	Lack of guidelines for biological testing
Extended drug circulation time	High production costs
Drug targeting (Increased drug concentration in target tissues)	Heterogenicity of diseases
Slowed down drug metabolism	Cytotoxicity
Drug accumulation	Variability of elimination and metabolism
Increased bioavailability	Complexity of dual drug-loaded or stimuli responsive nanosystems
Minimising side effects	Lack of reproducibility
Controlled and triggered release	Long-term stability
Combination therapy	Low clinical translation
Theranostic effect	Triggering of Immune response
Improving patient compliance	

**Table 3:** Mean zone of inhibition (mm) of different antibiotics with and without AgNPs against Gram-positive and Gram-negative bacteria * [117].

**Microorganisms**						
	**Erythromycin (10 μg/disk)**			**Kanamycin (10 μg/disk)**		
	**Zone (mm)**		**Fold increase %= ((b − a)/a) × 100**	**Zone (mm)**		**Fold increase %= ((b − a)/a) × 100**
	**Erythromycin (a)**	**Ag-NPs + Erythromycin (b)**		**Kanamycin (a)**	**Ag-NPs + Kanamycin (b)**	
*E. coli*	13	16	23.08	12	16	33.33
*S. typhi*	24	31	29.17	13	19	46.15
*S. aureus*	9	10	11.11	9	11	22.22
*M. luteus*	8	9	12.50	10	11	10.00
	Overall synergistic antibacterial effect (%)		18.96	Overall synergistic antibacterial effect (%)		27.93
**Microorganisms**						
	**Chloramphenicol (10 μg/disk)**			**Ampicillin (10 μg/disk)**		
	**Zone (mm)**		**Fold increase %= ((b − a)/a) × 100**	**Zone (mm)**		**Fold increase %= ((b − a)/a) × 100**
	**Chloramphenicol (a)**	**AgNPs + chloramphenicol (b)**		**Ampicillin (a)**	**AgNPs + Ampicillin (b)**	
*E. coli*	22	28	27.27	12	21	75.00
*S. typhi*	29	36	24.14	11	20	81.82
*S. aureus*	9	10	11.11	11	19	72.73
*M. luteus*	10	11	10.00	10	17	70.00
	Overall synergistic antibacterial effect (%)		18.13	Overall synergistic antibacterial effect (%)		74.89

* Note: Percentage fold increases in individual antibiotics were calculated using the formula ((b − a)/a) × 100. The mean zone of inhibition around disk containing AgNPs alone (10 μg) was 8 mm [117].

## Data Availability

Data sharing is not applicable to this article as no datasets were generated or analysed during the current study.
